# Feasibility and Safety of High‐Power Settings for Electrical Isolation of the Superior Vena Cava Using a Catheter Capable of Local Impedance and Contact Force Monitoring

**DOI:** 10.1002/joa3.70358

**Published:** 2026-05-07

**Authors:** Daisuke Kawano, Hitoshi Mori, Kei Matsumoto, Naomichi Tanaka, Tsukasa Naganuma, Wataru Sasaki, Masataka Narita, Kazuhisa Matsumoto, Yoshifumi Ikeda, Kazuo Matsumoto, Ritsushi Kato

**Affiliations:** ^1^ Department of Cardiology Saitama Medical University, International Medical Center Hidaka Japan

**Keywords:** atrial fibrillation, local impedance, radiofrequency ablation, superior vena cava isolation

## Abstract

**Introduction:**

Recently, catheters incorporating both contact force (CF) sensing and local impedance (LI) measurement—an indicator of tissue temperature—have become clinically available. The present study aims to evaluate the efficacy of superior vena cava isolation (SVCI) using this catheter and to investigate the characteristics of impedance during the procedure.

**Methods:**

This retrospective study included 64 patients undergoing initial SVCI using the StablePoint catheter (40 W, CF 10–20 g, 8–10 s). We analyzed the success rate of SVCI and impedance data collected from 1 402 ablation points, with particular attention to the dynamics of LI and generator impedance (GI).

**Results:**

First‐pass isolation was achieved in all cases without complications such as phrenic nerve or sinus node injury. The LI drop was significantly greater and faster than the GI drop in all segments. The 90% decay time of LI was consistently 5–6 s, suggesting effective lesion formation during the resistive heating phase. LI provides a more reliable and localized indicator of tissue response than GI. The temporal profile of LI supports its utility in guiding safe and effective lesion delivery, particularly in the thin myocardial tissue of the SVC.

**Conclusion:**

High‐power ablation guided by LI and CF using the StablePoint catheter enables safe and effective SVCI with accurate real‐time assessment of lesion quality.

## Graphical abstract

High‐power superior vena cava isolation using a contact force– and local impedance–sensing catheter achieved safe and consistent first‐pass isolation. Local impedance decreased more rapidly than generator impedance, accurately reflecting effective lesion formation and enabling reliable real‐time assessment in thin SVC myocardium.

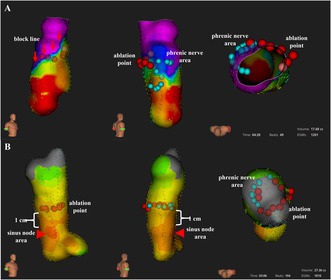

## 1 Introduction

Pulmonary veins (PVs) play an important role as the trigger and substrate for atrial fibrillation (AF), and PV isolation (PVI) is the cornerstone strategy for treating AF [1, 2, 3]. However, non‐pulmonary vein (non‐PV) foci also frequently contribute to AF initiation, with the superior vena cava (SVC) being one of the most common sites [4]. Therefore, performing electrical SVC isolation (SVCI) in addition to PVI may improve clinical outcomes of radiofrequency (RF) ablation for AF [5, 6]. In recent years, index‐guided ablation using parameters such as Ablation Index (Biosense Webster) and Lesion Index (Abbott) has been employed to create durable lesions, and their utility has also been reported in SVCI [7, 8, 9, 10, 11, 12]. However, these indices do not reflect tissue characteristics, making it difficult to predict complications such as steam pops [13]. Recently, local impedance (LI) technology, which reflects information about the tissue in contact with the catheter, has emerged as a valuable tool that contributes to safer ablation procedures. SVCI using catheters equipped with LI technology has been reported; however, these reports involve the IntellaNav MiFi OI catheter (Boston Scientific, Marlborough, MA, USA) [14], which lacks a CF sensor. To date, there have been no reports on SVC isolation using the IntellaNav StablePoint catheter (Boston Scientific, Maple Grove, MN), which incorporates a CF sensor, yet has a shorter tip length (Tip length: 4.0 mm) than the IntellaNav MiFi OI (Tip length: 4.5 mm). The aim of this study was to assess the efficacy of SVCI using this LI‐monitoring CF‐sensor catheter and to characterize impedance dynamics during the procedure.

## 2 Methods

### 2.1 Study Population

A total of 72 consecutive patients who underwent de novo SVCI using the StablePoint catheter during AF ablation at our institution between January 2021 and December 2022 were enrolled in this study. All patients underwent PVI, and additional ablation procedures, including cavotricuspid isthmus (CTI) ablation, posterior wall isolation (PWI), and mitral isthmus (MI) ablation, were performed as needed.

The study protocol was approved by the institutional review board of our hospital (2023–134).

### 2.2 Mapping and Ablation Protocol

All patients received either direct oral anticoagulants or warfarin for at least one month prior to the procedure. Antiarrhythmic drugs were discontinued for a minimum of five half‐lives before the ablation. Ablation procedures were performed under deep sedation with dexmedetomidine hydrochloride, propofol, and pentazocine. Following vascular access, heparin was administered at a dose of 100 U/kg body weight, and the activated clotting time was maintained at over 300 s throughout the procedure. A duodecapolar catheter (Map‐iT, ACCESS POINT, Rogers, MN, USA) was placed into the coronary sinus.

Mapping and ablation were performed under the guidance of a three‐dimensional (3D) mapping system (Rhythmia; Boston Scientific, Marlborough, MA, USA). A duodecapolar catheter (Map‐iT, ACCESS POINT, Rogers, MN, USA) was placed into the coronary sinus.

### 2.3 The Protocol of SVC Mapping

Electrical SVCI was selectively performed in patients with an SVC myocardial sleeve length > 1 cm, as determined by high‐density mapping, or in those who exhibited frequent premature atrial contractions originating from the SVC during isoproterenol infusion (0.06–0.10 μg/kg/min).

**FIGURE 1:**
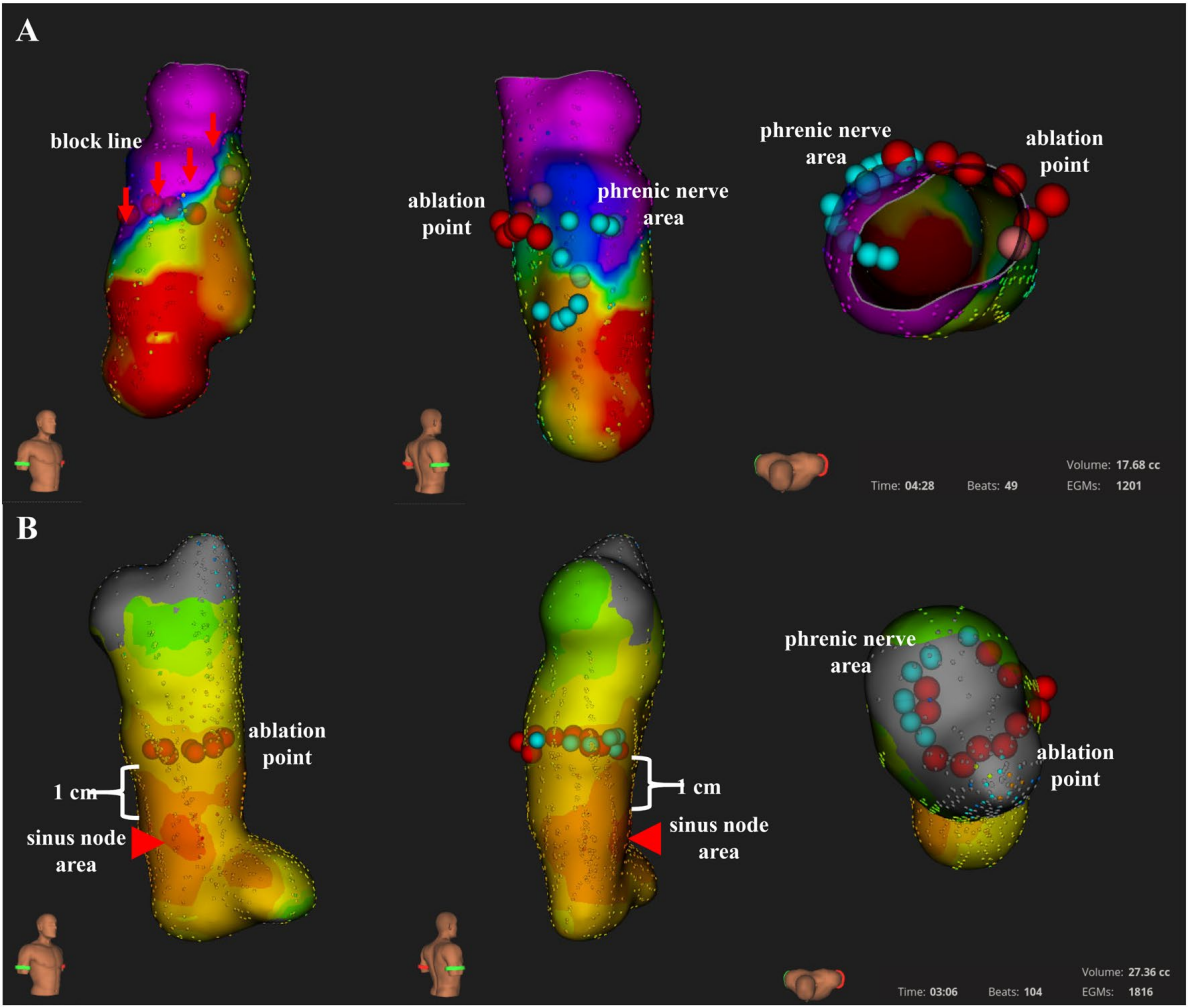
Ultra‐high resolution activation and voltage maps of the SVC and upper RA during sinus rhythm. In patients with a conduction block line (red arrow, upper panel), point‐by‐point RF delivery was performed circumferentially along the line connecting the open ends of the conduction block line. In patients without a conduction block line (lower panel), circumferential SVCI was performed at approximately 1 cm above the earliest activation site (white arrow). The red tags indicate the ablation sites. The blue tags indicate the sites where phrenic nerve stimulation was observed by high‐output pacing (10 V) on the posterolateral wall of the SVC. RA, right atrium; RF, radiofrequency; SVCI, superior vena cava isolation.

SVCI was performed using a 3D mapping‐guided approach, in which the block line was delineated on the electroanatomical map [15]. First, an electroanatomical map of the right atrium (RA) and SVC region was created during sinus rhythm using a high‐density 3D mapping system with a sixty‐four electrode mini basket mapping catheter (ORION; Boston Scientific, Marlborough, MA, USA). The RA–SVC junction was identified based on the anatomical reconstruction of the electroanatomical map and the abrupt change in electrogram characteristics between the RA and the SVC (Figure 1A). The sinus node region was identified as the site showing the earliest activation with a QS morphology on the unipolar electrogram (Figure 1, red arrow) [15]. Subsequently, pacing was performed near the presumed course of the phrenic nerve using a 10 V output from the ablation catheter to identify and avoid regions at risk of phrenic nerve injury (PNI). Regions where phrenic nerve capture was observed were documented and tagged on the electroanatomical map (Figure 1, blue points).

### 2.4 The Protocol of SVC Isolation

Point‐by‐point RF delivery was performed by using an open‐irrigated 4.0 mm tip electrode catheter (StablePoint catheter). For patients with an electrical block line at the RA‐SVC junction superior to the sinus node, ablation was performed on the posterior wall, avoiding the block line (Figure 1A). In the absence of a block line, ablation was performed at a site approximately 1 cm superior to the earliest activation site mapped near the sinus node (Figure 1B).

The myocardial thickness of the SVC is reported to be approximately 1–2 mm thick [16]. A previous study using the StablePoint catheter demonstrated that a lesion depth of 2 mm can be achieved with 40 W of RF energy in approximately 10 s [17]. Based on this approach, RF energy was delivered at 40 W with a CF of 10–20 g for 10 s per application. Interlesion distance was set to less than 6 mm. For ablation at sites where phrenic nerve capture was observed during pacing, RF energy was delivered under continuous fluoroscopic monitoring of diaphragmatic movement and immediately discontinued upon any reduction in diaphragmatic excursion.

The ablation endpoint of SVCI was defined as the complete elimination of all SVC potentials. Bidirectional block was confirmed by the absence of right atrial capture during SVC pacing from the ablation catheter. Dormant conduction was also assessed following intravenous administration of isoproterenol and adenosine triphosphate. We evaluated the rate of first‐pass isolation (FPI) and assessed the occurrence of procedure‐related complications associated with SVCI, including sinus node injury (SNI) and PNI.

### 2.5 Impedance Analysis

LI reflects the temperature of the tissue in contact with the catheter [18], and was measured between the entire distal tip (4‐mm tip) and the second ring electrode using the StablePoin catheter. Impedance drop (LI/GI drop) was defined as the difference between the impedance at the ablation start (initial LI/GI) and the impedance at the end of energy delivery. The percentage impedance drops (%LI drop and %GI drop) were also calculated by dividing the absolute drop by the corresponding initial values. Comparative analyses were conducted for initial impedance, impedance drop, and percentage impedance drop for both LI and GI.

LI and GI have been reported to correlate with tissue temperature and are known to decrease over time, with the rate of change gradually diminishing [18, 19, 20, 21, 22]. To assess the rate of change in LI/GI after the initiation of ablation, impedance measurements were recorded every second from the start of energy delivery until the termination of each application. The LI/GI drop and %LI/%GI drop at each ablation time were compared.

**FIGURE 2:**
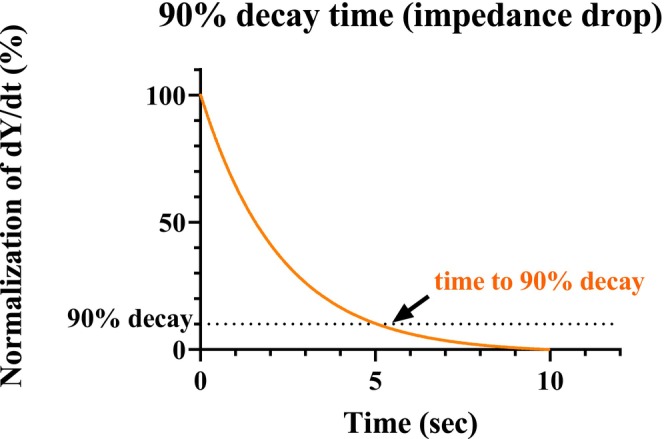
The analysis of the time to reach 90%. The rate of change in the time‐dependent change in the measured LI was assessed, and the time to reach a 90% decay in the peak dY/dT, where Y indicates the LI value, was expressed as the “time to a 90% decay”.

LI exhibits a rapid decline during the initial resistive heating phase immediately after the commencement of ablation (rapid phase), followed by a slower, more gradual decrease during the subsequent conductive heating phase (slow phase) [17]. Because the myocardial layer of the SVC is thin, excessive ablation may lead to collateral damage; therefore, shallow lesions primarily generated by resistive heating are considered sufficient for effective treatment. We previously reported that the time required for impedance to reach 90% of its final value at the end of ablation (90% decay time) reflects the resistive heating phase, which is characterized by rapid and active energy delivery. The rate of change in the time‐dependent gain of the measured LI was analyzed (Figure 2). The time to reach a 90% reduction from the peak of the first derivative (dY/dt), where Y denotes the LI value, was defined as the ‘time to 90% decay’ and was used for comparative analysis.

**FIGURE 3:**
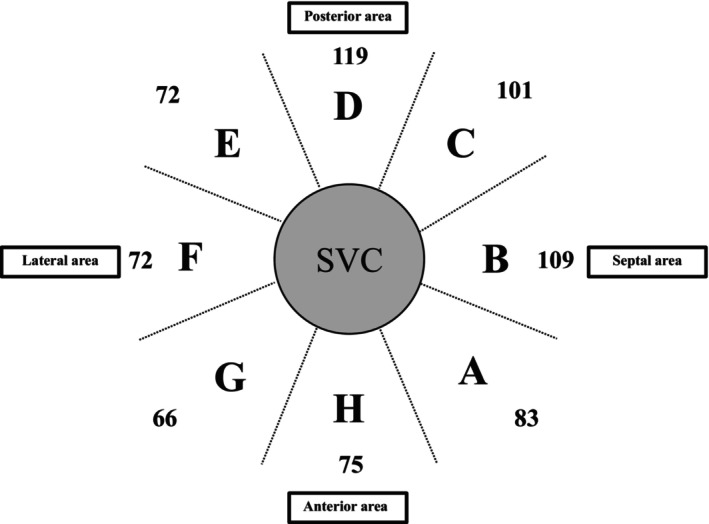
The ablation area of the SVC was analyzed. A total of 1 402 points were evaluated in this study. The superior vena cava (SVC) was circumferentially divided into 8 segments (A–H).

Impedance changes were analyzed by dividing the SVC into eight anatomical segments, and comparisons were conducted for each segment (Figure 3).

### 2.6 Statistical Analysis

The statistical analyses were performed using JMP software (version Pro16, SAS Institute, Cary, North Carolina) and GraphPad Prism 9 software (GraphPad Software Inc., San Diego, CA). Continuous data are expressed as the mean ± SD for parametric data or median (interquartile range [IQR]) for non‐parametric data. A comparison of the means between the groups was performed using an independent samples *t*‐test for normally distributed data and a Mann–Whitney U‐test for non‐uniformly distributed data. Categorical data were tested by aχ [2] test or Fisher's exact test. A value of *p* < 0.05 was considered statistically significant, unless specified otherwise.

## 3 Results

### 3.1 Patient Characteristics

Among the 72 patients, 8 were excluded from the analysis due to either undergoing SVCI with energy settings other than the standard 40 W (i.e., lower or higher power) or the unavailability of LI data related to the SVCI procedure. The remaining 64 patients were included in the analysis. Table 1 summarizes the baseline characteristics and procedural details. Paroxysmal AF was present in 71.9% of patients, while the remaining patients had persistent AF; no patients had long‐standing persistent AF. SVCI was performed in 13 patients during the de novo AF ablation session, in 43 patients during a second session, and in 8 patients during a third session. In all cases, PVI had been performed before SVCI. CTI ablation had been performed in 52 patients, PWI in 25 patients, and MI ablation in 2 patients during the current or prior ablation sessions.

**TABLE 1:** Baseline characteristics and the procedure details.

Baseline Characteristics (*n* = 64)	
Age, years	64.5 ± 11.3
Gender, Male, *n* (%)	36 (56.3)
BMI, kg/m^2^	23.7 ± 4.6
Paroxysmal AF, *n* (%)	46 (71.9)
Persistent AF, *n* (%)	18 (28.1)
Chronic Heart Failure, *n* (%)	10 (15.6)
Hypertension, *n* (%)	16 (25.0)
Diabetes, *n* (%)	7 (10.9)
History of Stroke, *n* (%)	5 (7.8)
Laboratory Findings	
BNP, pg/ml	27.8 (15.8–95.4)
Cr, mg/dl	0.92 ± 0.87
Echocardiographic findings	
LVEF, %	63.1 ± 11.4
LAD, mm	40.4 ± 7.3
Procedure details	
1st AF session, *n* (%)	13 (20.3)
2nd AF session, *n* (%)	43 (67.2)
3rd AF session, *n* (%)	8 (12.5)
Procedure time (min)	144.2 ± 56.8
Fluoroscopy time (min)	23.1 ± 13.0
Prior PVI, *n* (%)	64 (100)
Prior CTI, *n* (%)	52 (81.3)
Prior PWI, *n* (%)	25 (39.1)
Prior MI, *n* (%)	2 (3.1)
SVCI details	
SVCI time (min)	6.2 ± 4.6
Total number of RF applications	12.4 ± 4.0
Conduction block line, *n* (%)	38 (59.4)
Procedural Complications, *n* (%)	
Cardiac tamponade, *n* (%)	0
Thromboembolic events, *n* (%)	0
SNI, *n* (%)	0
PNI, *n* (%)	0
SVC stenosis, *n* (%)	0

*Note:* Continuous variables are shown as the mean (±SD) or median (first–third quartile) and categorical variables as the number (%).

Abbreviations: AF, atrial fibrillation; BMI, body mass index; BNP, brain natriuretic peptide; Cr, creatinine; CTI, cavotricuspid isthmus; LAD, left atrial diameter; LVEF, left ventricular ejection fraction; MI, mitral isthmus; PVI, pulmonary vein isolation; PWI, posterior wall isolation.

The mean ablation time for SVCI was 6.2 ± 4.6 min, with an average of 12.4 ± 4.0 RF applications. Conduction block between the RA and SVC was observed in 38/64 cases (59.4%). FPI for SVC was achieved in all cases without the need for touch‐up ablation or evidence of dormant conduction. No procedure‐related complications, including SNI, PNI, or SVC stenosis, were observed. Additionally, no steam pops occurred during any RF applications.

### 3.2 The Details of the Impedance Analysis at Each Location Segment

A total of 1 550 ablation points were delivered across 64 patients. Of these, 148 points were excluded due to one or more of the following reasons: (1) impedance rise; (2) missing data; or (3) RF delivery time less than 7 s. After these exclusions, 1 402 ablation points were included in the final analysis. The detailed distribution of RF applications across each anatomical segment is shown in Figure 3.

**FIGURE 4:**
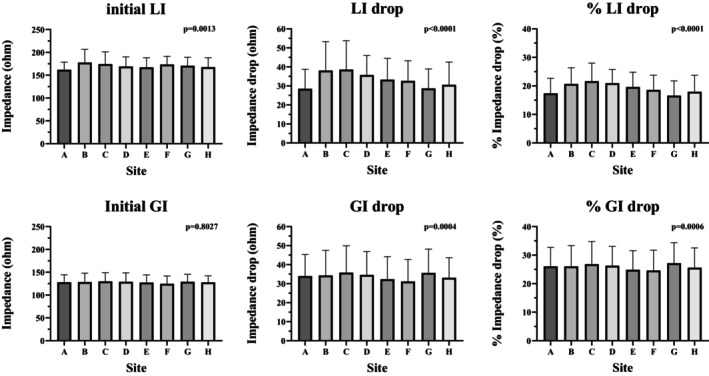
The details of the impedance at each location segment. This figure shows the details of the impedance at each location segment. All parameters except for the initial GI drop showed significantly different values at each location segment. The LI drop and % LI drop were highest at the posteroseptal area and lowest at the anterolateral area. On the other hand, GI drop and % GI drop were highest at the septal area and lowest at the posterolateral area (initial LI, ohm).

**FIGURE 5:**
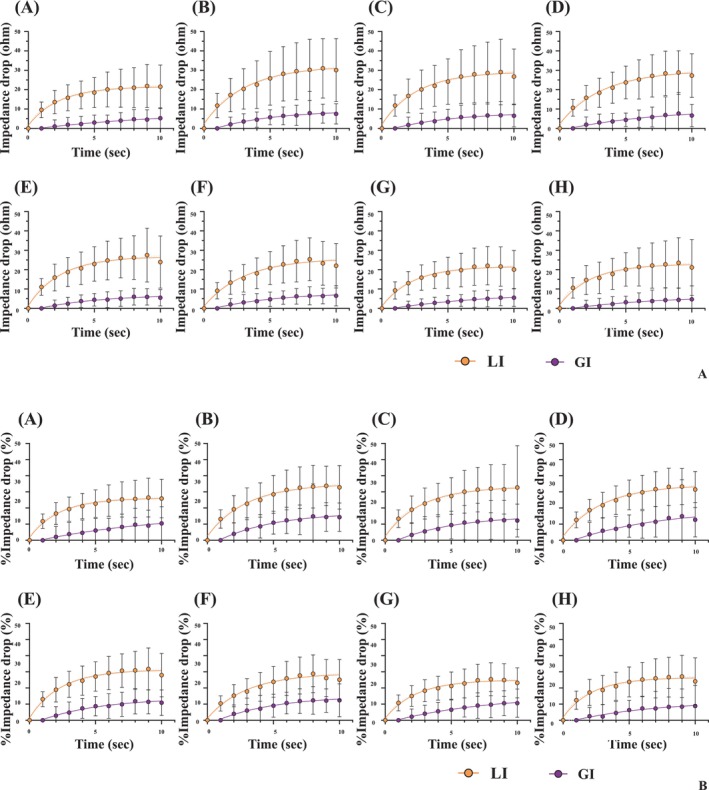
The comparison of the time course of LI‐related parameters and GI‐related parameters at each location segment of the SVC. This figure shows the comparison of the time course of LI‐related parameters and GI‐related parameters at each location segment of the SVC (A; impedance drop, B; %impedance drop). At all segments, the LI‐related parameters changed more gradually than the GI‐related parameters.

Figure 4 presents a comparison of the initial impedance, the absolute impedance drop, and the percentage impedance drop. The initial LI and GI were 161.9–177.5 Ω and 124.8–129.8 Ω, and the initial LI was significantly higher than the initial GI (*p* < 0.001). The impedance drop of the LI and GI during the applications was 28.5–38.6 Ω and 8.9–9.4 Ω, respectively, and the LI drop was significantly greater than the GI drop (*p* < 0.001). The percentage of impedance drop of the LI and GI during the applications was 16.6%–21.7% and 24.6%–27.1%, respectively, and the LI drop was significantly smaller than the GI drop (*p* < 0.001). The LI/GI drop and the % LI/GI drop varied at the ablation sites. LI drop and % LI drop were highest at the posteroseptal area and lowest at the anterolateral area. On the other hand, GI drop and % GI drop were highest at the septal area and lowest at the posterolateral area. At all segments, LI drop and % LI drop were significantly greater than GI drop and % GI drop (Table S1). Figure 5A,B show the temporal changes in LI and GI at each anatomical segment of the SVC. Both the absolute and percentage impedance drops were greater for LI than for GI from the onset of ablation. Table 2 presents the 90% decay time of the LI drop at all sites. In both absolute and percentage terms, the LI drop reached 90% of its final value within 5 to 6 s.

**TABLE 2:** Local impedance 90% decay time at each segment (s).

Location site	A	B	C	D	E	F	G	H
Impedance drop (s)	5.05	6.20	5.63	6.30	5.30	6.14	5.11	5.14
%Impedance drop (s)	4.93	6.03	5.69	6.10	5.13	6.04	5.09	4.87

## 4 Discussion

The major findings in our study were as follows:

1. 40 W for 8 to 10 s ablation was sufficient for the SVCI using the StablePoint catheter without any complications.

2. The value of the LI drop was larger than that of the GI drop at all SVC segments.

3. The 90% decay time of LI was reached within 5 to 6 s at all SVC segments.

### 4.1 Clinical Significance of Superior Vena Cava Isolation in Atrial Fibrillation Ablation

SVC is recognized as one of the non‐PV foci, and SVCI has been reported to contribute to the maintenance of sinus rhythm following catheter ablation [5, 6]. The randomized ESVCI‐AF trial did not demonstrate a significant benefit of empirical SVCI in addition to PVI, indicating that the clinical value of the empirical SVCI strategy remains controversial [23]. However, meta‐analyses have suggested that empirical SVCI in addition to PVI may reduce atrial arrhythmia recurrence without a significant increase in procedural complications, particularly in patients with paroxysmal AF [24, 25]. Importantly, SVCI may be beneficial in selected patients; therefore, in the present study, SVCI was not performed empirically but was selectively applied after initial PVI in patients with longer SVC myocardial sleeves or SVC‐related triggers. Accordingly, the present study does not support routine empirical SVCI. Instead, our findings provide technical and biophysical evidence supporting the feasibility and safety of local impedance–guided, high‐power SVCI when a trigger‐guided strategy is adopted. In this context, our results may contribute to optimizing selective SVCI techniques while minimizing procedure‐related risks.

More importantly, secondary factors such as hypertension, thyroid dysfunction, lifestyle‐related factors (including alcohol consumption and endurance sports), and underlying cardiomyopathies are known to influence AF burden [26, 27]. Optimal management of these upstream factors may reduce AF recurrence irrespective of the ablation strategy and may help re‐evaluate the indication for additional ablation procedures, such as SVCI, which are associated with non‐negligible procedural risks.

### 4.2 Safety and Efficacy of the 40 W Ablation Combining the High‐Density Mapping for SVC Using the StablePoint Catheter

The SVCI procedure carries potential risks of severe collateral damage, such as SNI, PNI, and SVC stenosis [4, 28, 29]. The wall thickness of the SVC has been reported as 1.2 ± 1.0 mm, which is thinner than that of the pulmonary veins (2.2–6.5 mm) and the cavotricuspid isthmus (CTI) (2.7–4.1 mm) [16, 30, 31]. Consequently, more cautious ablation strategies are required for SVCI, and several mapping and ablation techniques have been developed to minimize the risk of SNI and PNI.

High‐density mapping has shown utility in minimizing the risk of complications by identifying conduction block lines at the SVC‐RA junction, allowing for effective treatment with a reduced number of ablation lesions [15]. SVCI of this study was achieved using the FlexAbility catheter (Abbott Medical, Minneapolis, MN, USA) with relatively low‐power ablation at 20–30 W [15]. Due to the longer tip length of the StablePoint catheter, higher power settings are considered necessary [17]. In the present study, ablation was performed at 40 W with 8–10 s ablation, which enabled first‐pass isolation in all cases, regardless of the presence of a conduction block line. High‐power ablation is reported to be useful in less collateral damage due to reduced conductive heating [32, 33]. Consistent with this, no PNI was observed in the present study. In this study, to enhance procedural safety, several strategies were systematically employed, including high‐density mapping to identify functional conduction block lines, high‐output pacing to localize the phrenic nerve, continuous fluoroscopic monitoring of diaphragmatic movement, and high‐power RF delivery. Combining novel technologies with improved safety‐related technical advantages [34] and the strategies used in the present study may further lead to safer SVCI. Recently, pulsed field ablation (PFA) has been widely adopted in clinical practice [35]. Although PFA is considered to be highly myocardial selective and associated with a lower risk of neural injury, cases of phrenic nerve palsy following PFA have recently been reported [36]. In general, the current indications for PFA are limited to PVI and posterior wall isolation. Therefore, the present study is expected to contribute to the establishment of safe SVCI using RF energy.

### 4.3 Impedance Changes During the RF Application for the SVC Isolation

Impedance changes have been proposed as surrogate markers of electrode–tissue contact and lesion formation [19], and appear useful in predicting the creation of transmural lesions. GI has been shown to correlate with lesion size, but is affected by systemic factors such as body weight and lung interference, which can reduce its reliability. In contrast, LI reflects local factors—primarily the contacted myocardial tissue and adjacent blood—making it a more sensitive and reliable parameter [20, 21, 22]. In this study, while GI consistently decreased during ablation, both the magnitude and rate of decrease were smaller compared to those observed for LI (Figures 4 and 5). Furthermore, previous studies have demonstrated that LI drop is a better predictor of lesion formation than GI drop during PVI [21, 37]. Although the utility of LI drop with the MiFi catheter has been reported for SVC isolation [21], this study is the first to assess LI behavior during SVCI using the StablePoint catheter.

### 4.4 Time Course Analysis of LI Drop During SVC Isolation

Beyond the magnitude of the LI drop, its temporal profile may also provide important insights into lesion formation. In our study, the local impedance exhibited a characteristic pattern: A steep initial decline followed by a plateau phase. We analyzed the time required for the LI to reach 90% of its total drop (90% decay time), which reflects how quickly energy is delivered and tissue heating occurs. Interestingly, the rate of LI drop was found to decrease within the first 10 s of ablation (Figure 5), with the 90% decay time observed to be approximately 5 to 6 s. In this study, the ablation duration was set at 8 to 10 s, based on findings from prior ex vivo research [17]. This duration likely enabled lesion formation to be primarily driven by resistive heating, rather than conductive heating, as suggested by the rapid decline in LI. These findings imply that the current ablation protocol effectively targets resistive‐phase energy delivery, which may contribute to precise and controlled lesion creation.

## 5 Limitations

There are several limitations to our study. Firstly, there were no cases that underwent a 2nd session of SVCI. Thus, SVC reconnection was not evaluated, and the medium‐ to long‐term durability remains unclear. More recently, Mizunuma et al. have reported on the long‐term results of SVCI using high‐density mapping (CARTO system), comparing the block line group with the non–block line group [38]. In this study, there was no statistically significant difference in SVC reconnection between the two groups; however, the non–block line group exhibited a higher rate of SVC reconnections despite undergoing more extensive ablation. These findings might suggest that functional block line‐guided SVCI using high‐density mapping may provide durable isolation; however, this could not be confirmed in our study and warrants further investigation. Secondly, LI has been reported to reflect local tissue temperature and may be useful for predicting the occurrence of steam pops [21, 39]. In the present study, no steam pops were observed. However, the specific cutoff value of LI for predicting steam pops in the thin myocardial tissue of the SVC remains unclear. Finally, this study was a single‐center retrospective study with a relatively small sample of subjects. Further randomized controlled studies based on the SVCI with the StablePoint catheter would be needed to validate these findings in a larger cohort.

## 6 Conclusion

The 40 W for 8 to 10 s ablation with the StablePoint catheter was shown to be a feasible and safe procedure for SVCI with a high FPI rate and no complications. LI was superior to GI in reflecting tissue response and lesion formation, with a rapid LI drop and consistent 90% decay time of 5–6 s. These findings suggest that LI‐guided, resistive‐phase focused ablation may offer a safe and efficient strategy for SVCI.

## Author Contributions

D.K. and H.M., study conception and design; K.M., N.T., T.N., W.S., M.N., and K.M., data collection and data analysis; K.M., K.M., and R.K., manuscript revision; R.K. and K.M., study supervision.

## Funding

The authors have nothing to report.

## Ethics Statement

The study was approved by the institutional review board (2023–134).

## Consent

All patients provided their informed consent to the procedure and publication.

## Conflicts of Interest

The authors declare no conflicts of interest.

## Supporting information


**Table S1:** A comparison of the initial impedance, absolute impedance drop, and percentage impedance drop at each segment.

## Data Availability

The data that support the findings of this study are available on request from the corresponding author. The data are not publicly available due to privacy or ethical restrictions.
