# Supplementary material for: Feasibility and Safety of High‐Power Settings for Electrical Isolation of the Superior Vena Cava Using a Catheter Capable of Local Impedance and Contact Force Monitoring
Source: J Arrhythm. 2026 May 7;42(3):e70358. doi: 10.1002/joa3.70358 (PMC13150346; doi:10.1002/joa3.70358)
Supplement: Supplementary file 1 — Table S1: A comparison of the initial impedance, absolute impedance drop, and percentage impedance drop at each segment. [file JOA3-42-e70358-s001.docx]

| **Location site** | A | | B | | C | | D | | E | | F | | G | | H | |
| --- | --- | --- | --- | --- | --- | --- | --- | --- | --- | --- | --- | --- | --- | --- | --- | --- |
|  | LI | GI | LI | GI | LI | GI | LI | GI | LI | GI | LI | GI | LI | GI | LI | GI |
| Initial impedance  (ohm) | 161.9 | 128.1 | 177.5 | 128.5 | 174.2 | 129.8 | 169.1 | 129.0 | 167.3 | 127.3 | 173.4 | 124.8 | 170.8 | 129.2 | 167.6 | 127.9 |
| Impedance drop  (ohm) | 28.5 | 5.3 | 38.1 | 8.7 | 38.6 | 7.4 | 35.7 | 7.3 | 33.3 | 6.0 | 32.6 | 7.0 | 28.7 | 5.7 | 30.6 | 4.5 |
| %Impedance drop  (percentage) | 17.4 | 5.4 | 20.7 | 8.2 | 21.7 | 7.0 | 21.0 | 7.3 | 19.6 | 5.9 | 18.6 | 6.7 | 16.6 | 5.6 | 17.9 | 4.4 |

**Supplement Table. A comparison of the initial impedance, absolute impedance drop, and percentage impedance drop at each segment**

Abbreviations: LI, local impedance; GI, general impedance
